# The Phenotype and Treatment of WIP Deficiency: Literature Synopsis and Review of a Patient With Pre-transplant Serial Donor Lymphocyte Infusions to Eliminate CMV

**DOI:** 10.3389/fimmu.2018.02554

**Published:** 2018-11-02

**Authors:** Wolfgang Schwinger, Christian Urban, Raphael Ulreich, Daniela Sperl, Anna Karastaneva, Volker Strenger, Herwig Lackner, Kaan Boztug, Michael H. Albert, Martin Benesch, Markus G. Seidel

**Affiliations:** ^1^Division of Pediatric Hematology-Oncology, Department of Pediatrics and Adolescent Medicine, Medical University Graz, Graz, Austria; ^2^Pediatric Intensive Care Unit, Division of General Pediatrics, Department of Pediatrics and Adolescent Medicine, Medical University Graz, Graz, Austria; ^3^Ludwig Boltzmann Institute for Rare and Undiagnosed Diseases, Vienna, Austria; ^4^CeMM Research Center for Molecular Medicine of the Austrian Academy of Sciences, Vienna, Austria; ^5^Department of Pediatrics and Adolescent Medicine, Medical University of Vienna, Vienna, Austria; ^6^Department of Pediatrics, St. Anna Kinderspital and Children's Cancer Research Institute, Medical University of Vienna, Vienna, Austria; ^7^Dr. von Hauner University Children's Hospital, Ludwig Maximilian Universität, Munich, Germany; ^8^Research Unit for Pediatric Hematology and Immunology, Medical University Graz, Graz, Austria

**Keywords:** Wiskott-Aldrich syndrome protein-interacting protein (WIP), primary immunodeficiency (PID), Wiskott-Aldrich syndrome (WAS), hematopoietic stem cell transplantation (HSCT), donor lymphocyte infusions (DLI), combined immunodeficiency (CID) with syndromic features, inborn errors of immunity

## Abstract

Early diagnosis of primary immunodeficiency disorders (PID) is vital and allows directed treatment, especially in syndromes with severe or profound combined immunodeficiency. In PID patients with perinatal CMV or other opportunistic, invasive infections (e.g., *Pneumocystis* or *Aspergillus*), multi-organ morbidity may already arise within the first months of life, before hematopoietic stem cell transplantation (HSCT) or gene therapy can be undertaken, compromising the definitive treatment and outcome. Deficiency of Wiskott-Aldrich syndrome (WAS) protein-interacting protein (WIP deficiency) causes an autosomal recessive, WAS-like syndrome with early-onset combined immunodeficiency that has been described in three pedigrees to date. While WAS typically includes combined immunodeficiency, microthrombocytopenia, and eczema, the clinical and laboratory phenotypes of WIP-deficient patients–including lymphocyte subsets, platelets, lymphocyte proliferation *in vitro*, and IgE—varied widely and did not entirely recapitulate WAS, impeding early diagnosis in the reported patients. To elucidate the phenotype of WIP deficiency, we provide a comprehensive synopsis of clinical and laboratory features of all hitherto-described patients (*n* = 6) and WIP negative mice. Furthermore, we summarize the treatment modalities and outcomes of these patients and review in detail the course of one of them who was successfully treated with serial, unconditioned, maternal, HLA-identical donor lymphocyte infusions (DLI) against life-threatening, invasive CMV infection, followed by a TCRαβ/CD19-depleted, treosulfan/melphalan-conditioned, peripheral blood HSCT and repetitive, secondary-prophylactic, CMV-specific DLI with 1-year post-HSCT follow-up. This strategy could be useful in other patients with substantial premorbidity, considered “*too bad to transplant*,” who have an HLA-identical family donor, to eliminate infections and bridge until definitive treatment.

## Introduction

Wiskott-Aldrich syndrome (WAS) is an X-linked, classical combined immunodeficiency with syndromic features, defined by a clinical trias of immunodeficiency, eczema, and microthrombocytopenia that bears a risk of autoimmunity and lymphoma (1–5). Allogeneic hematopoietic stem cell transplantation (HSCT) is the treatment of choice, if a suitable donor is available. Deficiency of WAS protein-interacting protein (WIP) is a recessive disorder that leads to premature degradation of WAS protein (WASP) and thus highly resembles WAS. However, the clinical presentation and courses of previously reported patients with WIP deficiency were more heterogeneous than those of WAS patients (6–8), especially with regards to very early onset of severe, life-threatening immunodeficiency. The conditioning pretreatment before HSCT of patients with WAS and other profound combined immunodeficiencies includes strongly immunosuppressive chemotherapy, and the outcome depends on the degree of preexisting and acquired invasive infections until engraftment enables the generation of an intact immune system.

## Functional aspects of WIP or WASP deficiency

WASP is present in the cytoplasm of hematopoietic cell lineages in an auto-inhibited, inactive form that is stabilized in a chaperone-like fashion by WIP binding (9). Upon activation by cell division cycle 2 (CDC42), a Rho family GTPase, activated by a variety of transmembrane receptors, or by proline-serine-threonine phosphatase-interacting protein 1 (PSTPIP1), and phosphatidylinositol-4,5-biphosphate (PIP2), the inhibitory, intramolecular, interaction is released, and actin polymerization is initiated [reviewed in Cotta-de-Almeida et al. (10)]. Besides the WASP-stabilizing function of WIP, it was shown to facilitate WASP activation by recruiting WASP to the immunological synapse upon activation of the T cell receptor and by enhancing CDC42-dependent activation (11). Actin polymerization is necessary for the immunological synapse formation, its stability, intracellular signaling, cytokine secretion, cellular migration, and lytic granule polarization, but there are also other actin-independent effects of WASP such as activity as transcription factor for T-bet and as anti-apoptotic survival factor (10). In addition to the T cell pathology notoriously associated with WAS, many of these functional defects also affect B cells [such as migration toward a chemokine gradient (8)] and NK cells (12, 13), and there is evidence from murine studies, that WIP has WASP-independent effects on B cell function, being a regulator of CD19 activation and PI3K/Akt signaling in B cells (14). Active WASP is stabilized by tyrosine phosphorylation, and its activity is counter-balanced by ubiquitin-dependent proteasomal degradation [reviewed in Cotta-de-Almeida et al. (10)].

## Synopsis of six WIP-deficient patients from three pedigrees

The first description of a WIP-deficient patient was from a girl from Morocco in 2012, who had been diagnosed and successfully transplanted with cord-blood-derived stem cells in Brescia, Italy, at 4.5 months and followed-up at that time for 16 months (6) (Table 1, pedigree A). Her appearance largely resembled WAS, but at that time, except for rare cases of imbalanced X chromosomal inactivation (15, 16), WAS in females or a recessive form was unknown or genetically unresolved (17). Results of immunological analyses, including the T and NK cell functions and an absence of WAS-protein despite normal *WAS* DNA and mRNA sequences, lead physicians to suspect degradation of WAS-protein due to WIP deficiency (6). Four years later, a large, branched family in Saudi Arabia was reported in which four (of 12 affected) individuals with WIP deficiency were described (7) (Table 1, pedigree B). Of these four patients, two underwent HSCT from a matched sibling and two from unrelated cord blood; one patient died shortly after HSCT from CMV-mediated, respiratory distress syndrome (7). The platelet phenotype varied inter-individually between normal-sized or microplatelets and moderate to severe thrombocytopenia (Table 1). Similarly, skin lesions varied, but an increased risk of infections, chronic diarrhea, and respiratory symptoms were constantly observed. The diagnosis of these patients was established by panel sequencing. The sixth patient was described in a report on the effect of WIP deficiency on the actin cytoskeleton and functional lymphocyte architecture recently (8); and a detailed clinical description with longer follow-up of this patient is provided below and in Table 1 (pedigree C). All hitherto-described patients displayed an extremely early onset of symptoms of primary immunodeficiency, but varying syndromic and laboratory features (Table 1).

**Table 1:** Clinical synopsis of WIP deficiency.

	**Pedigree A**	**Pedigree B**	**Pedigree C**
Publication	(6)	(7)	(8)
Ethnic/geographic origin	Morocco	Saudi Arabia	Kurdish/Turkish
Consanguinity	Yes	Yes	Yes
Number of described individuals/families	1 Female; another previously deceased sibling suspected/1 family	12 Affected, 4 of whom described/1 branched family	1 Male/1 family
Centre	Brescia, Italy	King Faisal SHRC, Riyadh, Saudi Arabia	Graz, Austria
Genetic aberrationMethod of detection	c.1301 C>G stop-gain Sanger sequencing after Western blot for WASP	c.709 C>T stop-gain whole exome & Sanger sequencing	c.373 G>A stop-gain next generation sequencing panel & Sanger sequencing; + cgh array to exclude chromosomal aberrations
Detection of WASP	Undetectable	Not reported	Undetectable
Gestational age, Birth length, weight, head circumference	No abnormalities reported	No abnormalities reported	Normal: 42 weeks, 49 cm (25%), 3,340 g (25–50%), 36.5 cm (75%)
Onset of symptoms (age)	11 days	1–8 weeks	4 weeks
Head and neck face, ears, eyes, nose, mouth	Oral ulcerations on hard palate and tongue (stopped after cord blood transplantation [CBT])	Persistent ear infection (*n* = 1)	Recurrent middle ear infections, large ears and eyes, slightly downward-slanted palpebral fissures, prominent forehead, nose, and philtrum
Cardiovascular	No abnormalities reported	No abnormalities reported	No abnormalities reported
Pulmonary	Respiratory distress syndrome (RSV), requiring ICU treatment, red cell and platelet transfusions, antibiotics	CMV pneumonitis (*n* = 3), respiratory infections (*n* = 4)	Viral bronchiolitis of unknown specificity, CMV pneumonitis requiring ICU treatment with long-term mechanical ventilation; obstructive bronchitis
Abdomen	Rotavirus enteritis, hepatopathy of unknown etiology	Chronic diarrhea (*n* = 4); bloody stools (*n* = 3); hepatosplenomegaly (*n* = 1)	Inguinal hernia, recurrent bloody diarrhea, suspected recurrent intussusception, intestinal volvulus and near-total small bowel resection; hepatosplenomegaly
Genitourinary	No abnormalities reported	No abnormalities reported	No abnormalities reported
Skeletal	No abnormalities reported	No abnormalities reported	Postnatal growth delay
Skin, nails hair	Eczematous rash and papulovesicular lesions on the scalp; The deceased sibling was reported to have suffered from ulcerative and vesicular skin lesions	Scaly eczematous skin lesions (*n* = 2), alopecia (*n* = 1)	No abnormalities reported
Neurologic	No abnormalities reported	No abnormalities reported	Delayed development during ICU admission, but catch-up development after clearance of infections
Endocrine	No abnormalities reported	No abnormalities reported	No abnormalities reported
Immunology, main features	T cell lymphopenia (800 CD3/μl), CD8+ relatively lower than CD4+, normal TCRγδ+ T cells, Borderline low B cells (300/μl), Increased NK cells (2,500/μl), IgG, A, M normal, IgE increased (32 IU/ml) T cell proliferation upon PHA normal, but reduced upon CD3 stimulation	Total T cell count normal, but mild reduction of CD4+ T cells (in 3 of 4 patients) and severe reduction of CD8+ T cells (*n* = 4), Normal B cells, variable NK cell number IgG, A, M normal or increased IgE increased (327–1,994 kU/L, determined in 2 patients) T cell proliferation upon PHA normal (*n* = 4)	Total T cell count normal, but severe reduction of naïve CD4+ T cells (CD4+CD45RA+ decreasing from 200 to 60/μl at 4-5 months of age; 1.86% TREC in CD3+CD45+) and rather increased CD8+ T cells (attributed to CMV infection), substantially increased proportion of TCRγδ+ T cells (>50% of CD3+; [normal <15%]), Normal B cells, Normal NK cells IgG and IgM increased, IgA normal, IgE increased (192–1,300 IU/mL) T cell proliferation upon PHA reduced, mildly reduced upon ConA, low-normal upon CD3/CD28
Other laboratory abnormalities	Moderate thrombocytopenia with normal platelet volume (average 59,000/μl platelets)	Moderate thrombocytopenia (*n* = 3, 75,000/μl) or severe thrombocytopenia (*n* = 1, 26,000/μl) with normal (*n* = 3) or reduced (*n* = 1) platelet volume	Mild-moderate thrombocytopenia (80-150,000/μl); later in course severe thrombocytopenia (30–40,000/μl even under romiplostim) with reduced platelet volume (≤8fl; confirmed microscopically)
Other (phenotypic) features	Failure to thrive, No autoimmunity or bleeding tendency reported	Not reported	Unspecific syndromic facial dysmorphia, No clinically manifest autoimmunity reported
Treatment	CBT at 4.5 months of age; reported at 16 months follow up clinically healthy with complete T and B cell donor chimerism but mixed myeloid and NK cell chimerism.	MSD-HSCT (*n* = 2; one died from CMV-RDS shortly post-transplant) and CBT (*n* = 2); three surviving patients are well 2–12 years post-transplant	Sequential maternal HLA-identical donor lymphocyte infusions successfully cleared CMV and other infectious problems when clinical condition was too bad for preparative chemotherapy for HSCT, chronic severe microthrombocytopenia persisted; needed IgG supportive substitution during ICU stay but not since DLI treatment; conditioned HSCT at 2.5 years of age was successful, restoring immune system and platelet counts; alive and well 1 year post-HSCT.

*WASP, Wiskott-Aldrich syndrome protein; CBT, cord blood transplantation; cgh, comparative genomic hybridization; RSV, respiratory syncytial virus; ICU, intensive care unit; CMV, cytomegalovirus; TCR, T cell receptor; TREC, T cell receptor excision circles; PHA, phytohemagglutinin; ConA, concanavalin A;MSD-HSCT, matched sibling donor hematopoietic stem cell transplantation; CMV-RDS, cytomegalovirus-mediated respiratory distress syndrome; DLI, donor lymphocyte infusions. The left and center columns summarize clinical and laboratory findings in previously published patients(6, 7), the right columns shows data from the patient presented in this report and, in part, in Pfajfer et al. (8)*.

**Figure 1:**
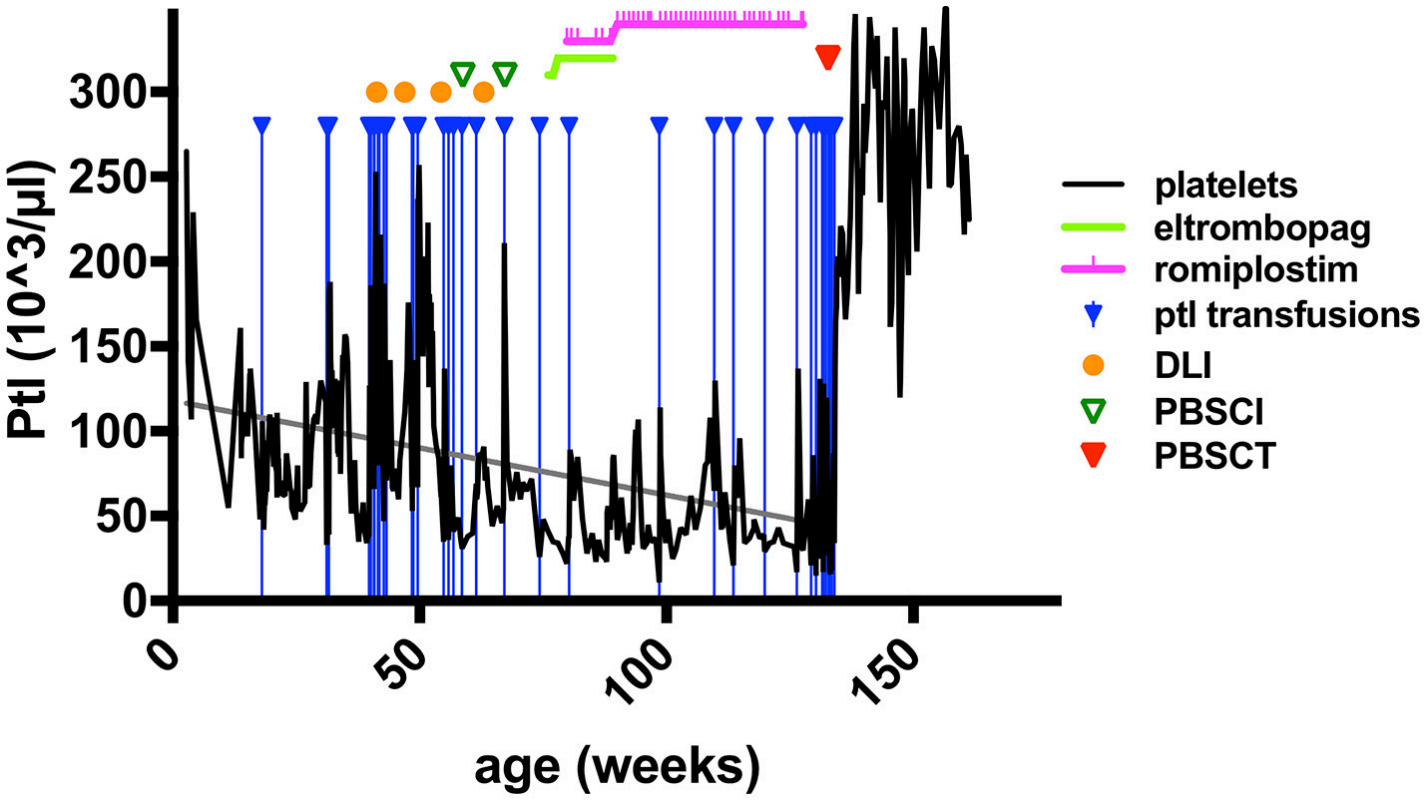
Platelet counts and therapeutic interventions in a patient with WIP deficiency. The course of platelet counts of the patient of pedigree C (Table 1, right column) is shown as black solid line (x1,000/μl) in weeks of life (x-axis). Treatment interventions are shown in the figure legend as indicated. Doses of cell transfer and stem cell transplantation are stated in the main text. Ptl, platelets; DLI, donor lymphocyte infusions; PBSCI, peripheral stem cell infusion (without prior conditioning treatment); PSCT, peripheral stem cell transplantation (HSCT after conditioning pretreatment as described in the main text).

As described briefly previously, the sixth WIP-deficient patient in this cohort (pedigree C, Table 1) became symptomatic with viral bronchiolitis 4 weeks after birth, required hospital admission and mechanical ventilation for pneumonitis at 3 months, due to a systemic CMV infection (detectable in plasma, urine, stool, saliva, bronchial secretion, cerebrospinal fluid) (8). Immunoglobulin-G was needed to be substituted intravenously. Platelet concentrates were given sporadically for intermittent bloody diarrhea with progressive thrombocytopenia (Table 1; and Figure 1), suggesting the presence of a relapsing intussusception, inflammatory bowel disease, or both. Endoscopy was performed at 6 months of age and biopsies showed signs of chronic erosive focal duodenitis (similar to infection but without detection of CMV or Cryptosporidium), further, a chronic, active gastritis and duodenitis (resembling reflux-association) were found. At that time, the colon was normal, and CMV, HSV, or other microorganisms could not be detected *in situ*. At 9 months of age, he had another endoscopy which showed active duodenitis with CMV-positive stroma cells and mild colitis with lymphoplasmacellular and patchy neutrophil infiltrations, but no microorganisms or viruses detectable despite CMV nucleic acid positivity in many body fluids at earlier time points. An acute clinical deterioration was observed at 11 months of age accompanied by clinical signs of intussusception. Emergency surgery was performed and necrotic bowel was resected, showing perforations, erosions, and ulcerations, as well as peritonitis. The mucosa could not be evaluated due to necrosis, the surgery yielded a severe short bowel syndrome. His psychomotor development was found to be impaired, despite displaying no signs of encephalitis (imaging; EEG; clinical) or meningitis (CSF; imaging). However, after 25 days of invasive and 94 days of nasal CPAP ventilation, his neurological status was difficult to assess.

The results of immunological analyses performed at 4 and 5 months of age showed clear signs of combined immunodeficiency with reduced naïve T cells, reduced T cell proliferation *in vitro*, and an increased proportion of T cell receptor gamma-delta (TCRγδ)-positive T cells (Table 1). Because ganciclovir treatment did not efficiently eliminate CMV, the clinical situation was considered too unstable for a conditioned HSCT. Since the mother was HLA-identical and CMV-seropositive, an attempt of maternal DLI (1 × 10^6^/kg CD3+ T cells) was performed in his 10th month of life. Previous reports had shown the long-term efficacy of unconditioned DLI in the setting of a patient series with 22q11 syndrome, ZAP70 deficiency, and other syndromes (18, 19). The first DLI was given under sirolimus to prevent the onset of graft-vs. host disease (GvHD), the following DLIs were administered without immunosuppression, and all were tolerated well. This led to clinical improvement and a gradual reduction in the CMV plasma load, with complete clearance after six doses (four DLI at 1 × 10^6^-1x10^7^/kg CD3+ cells and two peripheral stem cell concentrates at 2 × 10^7^/kg CD34+ plus 2 × 10^7^/kg CD3+ cells), and a stable mixed T cell donor chimerism over >12 months (>50–70% donor T cells), but expectedly did not lead to the engraftment of myeloid cells (chimerism of CD33+ cells <2–3% in peripheral blood) (8). Meanwhile, findings from NGS panel diagnostics led to the diagnosis of WIP deficiency due to the identification of a novel, stop-gain mutation in *WIPF1* [c.C373T, p.R125X; Table 1 and Pfajfer et al. (8)].

There were three indications to proceed to full conditioning and HSCT after the elimination of CMV in this patient: (i), an increasing frequency of ear-nose-throat (ENT) and respiratory infections despite the predominant donor T cell chimerism and IgG substitution; (ii), the uncertainty about the durability of the DLI-mediated T cell engraftment; and, (iii), most urgently, the continuous decline in thrombocyte counts (Figure 1). The latter did not sustainably respond to thrombopoietin receptor agonists, as treatment with eltrombopag had been attempted without effect due to suspected malresorption in short bowel syndrome, and even a switch to romiplostim was ineffective, although both substances were given at standard and increased dose intensities (Figure 1). Consequently, the patient was dependent on platelet transfusions. Furthermore, the psychoneurological situation had improved substantially, indicating that severe brain damage may not have occurred. The transplantation was carried out from his mother after conditioning with ATG-Grafalon (3 × 10mg/kg; days −8, −7, −6), treosulfan (3 × 12g/m^2^; days −5, −4, −3), and melphalan (140 mg/m^2^; day −2) at the age of 2.5 years, 15 months after receiving the last DLI and peripheral stem-cell infusion. The graft contained peripheral TCRαβ/CD19-negatively selected cells (5.7 × 10^7^/kg CD34+ and 1.9 × 10^7^/kg TCRγδ+CD3+ cells), and a second fraction of CD34+ positively selected stem cells (7.2 × 10^6^/kg CD34+ and 2.2 × 10^4^/kg CD3+ cells), providing high numbers of stem cells to facilitate rapid engraftment. GvHD prophylaxis consisted of mycophenolate mofetil (1,200 mg/m^2^), administered until day +46. Engraftment was excellent with 35,000/μl leukocytes on day +8; platelet transfusion was last required on day +9, with persisting full donor chimerism. Ganciclovir was administered from day −10 until day +53 as a secondary prophylaxis. In addition, five doses of CMV-specific DLI were given until day+84 in 2–4-weekly intervals to prevent CMV reactivation. CMV nucleic acid was detectable despite these measures in low-copy numbers (1 × 10^2^-1.5 × 10^3^/mL blood) without clinical correlates until day +90, at which point it was again negative. Apart from experiencing challenging alimentation problems due to the short bowel syndrome and a transient, acute, toxic pancreatitis, the patient did not experience any severe complications. He has been progressing wonderfully, and parenteral nutrition was gradually reduced until it was provisionally terminated at 9 months post-HSCT. He is currently well with >12 months follow-up after conditioned HSCT.

Because WASP undergoes proteasomal degradation, proteasome inhibition in a condition with unimpaired WASP synthesis could hypothetically result in intracellular stabilization of WASP and functional restoration even in WIP deficiency. Therefore, we performed an extensive *in vitro* screen with various proteasome inhibitors, including bortezomib, with inconclusive results. Finally, proteasome inhibition *in vivo* was not attempted in any WIP deficient patient reported so far.

## Comparison of the human phenotype with mice deficient in WIP

Newborn and young WIP null mice did not display a pronounced phenotype, neither anatomically, developmentally, nor hematologically, or immunologically (20, 21). However, after 8–10 weeks of life, granulocytosis and T- and B cell lymphopenia developed, splenomegaly was noted, while platelet numbers and volume were normal (21). A progressive lymphopenia was noted in lymph nodes and Peyer's plaques. Most prominently in the clinical phenotype, mice suffered from autoimmune inflammatory disease in many organs such as the gut, lungs, kidneys, joints, but not skin or other organs; and antinuclear and anti-cytoplasmatic antibodies were detected (21). The reported differentiation block, signaling and functional alterations of lymphocytes in WIP null mice including synapse formation and CD19 malfunction (14, 20) associated with autoimmunity and inflammation is similar to what was observed in humans with WIP deficiency. However, the degree of immunodeficiency in humans was not reflected by mice under laboratory conditions of animal facilities, and the platelet and megakaryocyte phenotype, which is variable in humans with WIP deficiency but rather constantly seen in WASP deficient humans and mice (1, 22), was not reported in WIP-deficient mice at all (21).

## Discussion

We provide a comprehensive synopsis of clinical and laboratory/biological features of all hitherto-published patients with WIP deficiency. Highly resembling WAS, subtle differences in presentation of WIP deficiency can be observed in addition to the obvious difference that stems from the autosomal recessive pattern of inheritance. The thrombocytopenia in WIP deficiency seems to be less severe and inconstant, and microthrombocytopenia is less frequent than in WAS, raising the question whether other molecules could substitute for WIP as chaperones for WASP specifically in platelets and megakaryocytes. This might be supported by the absence of thrombocytopenia or microplatelets in WIP deficient mice, as reported so far. It is striking that the T-cell immunodeficiency presents quite early in WIP deficient patients, while even classic WAS patients often don't present with severe opportunistic infections before the age of one. The patient reviewed in more detail here (the sixth of all previously published patients; from pedigree C) displayed nearly absent naïve CD4 T-cells and TRECs, which was not tested in the other patients, and had reduced T cell proliferation upon stimulation with PHA, a pathology not detected in previously tested individuals. This and the fact that maternal T-cells were able to engraft without conditioning may implicate a more severe T-cell deficiency than is commonly found in WAS. On the contrary, we cannot explain why in that patient T cell proliferation was tested low-normal upon stimulation with CD3/CD28 in contrast to a previously tested individual (from pedigree A). For these reasons WIP deficiency will be more difficult to diagnose. It does suggest that all infants with clinically severe T-cell deficiency, especially if accompanied by thrombocytopenia, should be tested for WIP deficiency.

Given the severe T-cell deficiency, early diagnosis of WIP deficiency seems prudent. Certainly, newborn screening (NBS) for TRECs would have accelerated the diagnosis in our patient [reviewed in Kwan and Puck (23)]. However, patients with combined immunodeficiency might not always be detected by TREC-based NBS with cutoffs designed to detect SCID, and NBS is not globally available. At the time of PID diagnosis, the patient from pedigree C showed many contraindications for conditioned HSCT, such as a systemic, invasive CMV infection, neurological impairment, and others. Similar to previous observations (18, 19), the patient had an HLA-identical, and, in his case, CMV-competent and WIP-proficient, family member. Despite the risk of GvHD, the potential anti-infectious activity of peripheral blood lymphocytes from such a donor, even with long-term “repopulating” capacity (18, 19), was an attempt to stabilize the clinical condition and buy time. Together, this clinical observation (8) indicated that, also for other patients with SCID or profound CID, an HLA-identical lymphocyte donor might be identified, especially, in highly consanguineous pedigrees, and a patient in a condition that is “*too bad to transplant*” might be rescued by taking a DLI approach and bridging to a subsequent HSCT.

## Ethics statement

The study was performed with informed consent and according to an IRB approval (24-334 ex 11/12; IRB00002556) in accordance with the Declaration of Helsinki.

## Author contributions

WS, CU, MB, RU, AK, DS, HL, VS, and MGS cared for the patient and collected the data. MGS wrote the manuscript, and designed table and figure. MB, MA, and KB analyzed and interpreted clinical and laboratory/genetic data, respectively, and corrected the manuscript.

### Conflict of interest statement

The authors declare that the research was conducted in the absence of any commercial or financial relationships that could be construed as a potential conflict of interest.

## Abbreviations

WASP: Wiskott-Aldrich syndrome protein
WIP: WASP interacting protein
PID: primary immunodeficiency
NGS: next generation sequencing
DLI: donor lymphocyte infusions
CMV: cytomegalovirus.
